# Sociodemographic, health-related, and external determinants of quit attempts among adult tobacco users: A cross-sectional study using a nationally representative sample in Oman

**DOI:** 10.18332/tpc/209456

**Published:** 2025-10-24

**Authors:** Salma R. Al-Kalbani

**Affiliations:** 1 Directorate General of Health Services, Muscat, Ministry of Health, Muscat, Oman

**Keywords:** tobacco cessation, quit attempts, smoking behavior, quit advice, Oman, cross-sectional study

## Abstract

**INTRODUCTION:**

Tobacco use is a global epidemic, with two out of three smokers trying to quit. In Oman, little progress has been made in implementing the WHO FCTC best practice recommendations. This study aims to examine the determinants of quit attempts among tobacco users in Oman.

**METHODS:**

A cross-sectional study was conducted using secondary data from the STEPS, Oman, 2017. Descriptive and bivariate analyses were performed initially. Binary logistic regression analysis was performed to examine the association between quit advice and quit attempt (the primary outcome of interest), after adjustments for possible confounders.

**RESULTS:**

Quit attempts were significantly higher among tobacco users with higher level of education (AOR=3.35; 95% CI: 1.67–6.72, p<0.0001), older age groups (AOR=1.77; 95% CI 1.00–3.13, p=0.048), and higher income groups (AOR=2.21; 95% CI: 1.24–3.93, p=0.007), compared to their counterparts. After adjusting for possible confounders, receiving advice from a healthcare worker to quit tobacco products was associated with a 3.13 times higher likelihood of attempting to quit (AOR=3.13; 95% CI: 1.46–6.71, p=0.003). Participants who had seen a health warning on television were 2.06 times more likely to attempt to quit compared to those who did not see one (AOR=2.6; 95% CI: 1.02–4.12, p<0.043).

**CONCLUSIONS:**

This study indicates a socioeconomic disparity in quit attempts, with higher rates observed among older adults, individuals with higher level of education, employed individuals, and those with higher monthly income. Quit advice provided by healthcare professionals was significantly associated with more quit attempts. These results highlight the importance of national-level coordination and monitoring to enhance the effectiveness of tobacco cessation program as part of comprehensive multi-sectoral tobacco control efforts. Further research is warranted to explore the broader determinants of quit attempts in Oman.

## INTRODUCTION

Tobacco use is a global epidemic that causes deaths of half of its users^1^. Its effects are unequally distributed among nations, with low- and middle-income countries suffering more than high-income countries^1,2^. The effects are also unevenly spread within countries, with more disadvantaged groups experiencing greater harm than less disadvantaged groups^2^. Tobacco use is linked to various health issues, including cancers, heart diseases, lung diseases, type 2 diabetes mellitus, premature birth, and sudden infant death syndrome^2^.

Tobacco use in Oman has increased in recent years, reaching 8% in 2020, with a large sex discrepancy, 15.5% among males and 0.4% among females, raising concerns about the short- and long-term health consequences^3^. According to the 2019 Global Burden of Disease study, tobacco use – which includes smoking, chewing tobacco, and exposure to secondhand smoke – was responsible for 1077 deaths (8.7% of all fatalities) in Oman; the majority of these deaths occur in people aged ≥50 years^4^. The highest disability-adjusted life year (DALY) burden attributed to tobacco use was observed in ischemic heart disease (26.27%), aortic aneurysms (34.54%), and peripheral artery disease (25.28%). Specifically, tobacco products accounted for approximately 11.5% of all diabetes mellitus deaths and nearly 12.0% of diabetes-related DALYs. In 2016, the total monetary cost of smoking and secondhand smoke in Oman was $637 million, representing over 1% of the country’s GDP^5^. This imposes additional strain on the healthcare system, which is already burdened with non-communicable diseases that are largely preventable through comprehensive strategies targeting their root causes.

Nicotine is addictive, possibly much more than the addictions caused by several illegal substances^6^. Nicotine addiction is highly influenced by factors such as the quantity and frequency of use, additives in tobacco products, peer pressure, stress, and environmental exposure^6-8^. Although two out of every three smokers attempt to quit, most of these attempts fail due to withdrawal symptoms^6-8^. The likelihood of making a quit attempt and successfully quitting is influenced by both individual factors and broader social determinants of health^7,9,10^. Withdrawal symptoms, which may begin one to two days after quitting and peak within the first week, can occur for all forms of tobacco products, both smoked and smokeless^11^. These symptoms include stress, increased appetite, depression, difficulty concentrating, irritability, and sleep disturbances^11^.

In Oman, nearly half of adult males and two-thirds of adult females reported having attempted to quit tobacco products in the past 12 months preceding the STEPS survey^12^. However, one-third of current smokers (37.7%) reported receiving advice to quit tobacco products by healthcare personnel, with males being twice as likely as females to receive such advice^12^.

Article 14 of the WHO Framework Convention on Tobacco Control (FCTC) obligates parties to design and implement effective program to promote tobacco cessation and to provide comprehensive guidelines for cessation support^13^. However, only one-third of the world’s population has access to effective tobacco cessation services^9^. Many governments have failed to provide comprehensive, accessible tobacco cessation therapy, often due to financial constraints or the belief that people are to blame for their addiction; however, both assumptions are incorrect^9^.

In Oman, no significant progress has been made in implementing the best practice recommendations by the WHO FCTC, including having a national toll-free Quitline, cessation counseling, and cessation medication^9^. Tobacco cessation services remain limited to primary healthcare settings, and they frequently encounter issues such as staff shortage and medication availability^14^. Brief advice is not routinely provided at each patient encounter. Moreover, little attention has been given to identify the social determinants of tobacco users’ attempt to quit. The purpose of this study is to examine the sociodemographic and health-related characteristics associated with quit attempts among tobacco users in Oman, with a specific focus on the association between receiving quit advice from healthcare workers and reported quit attempts.

## METHODS

### Study population

The population in this study consists of adult tobacco users aged ≥18 years who participated in the STEPS 2017 survey in Oman^15^. The primary survey was a nationally representative community survey, which was conducted based on the WHO STEPwise approach to surveillance (STEPS). It used a multi-stage cluster sampling procedure and was carried out using face-to-face interviews, with anthropometric measurements taken by qualified staff. The total number of participants was 6743 (response rate was 97.9%), of whom only 512 were tobacco users, accounting for 7.6% of study participants^15^.

### Ethical approval

This study was based on secondary data from STEPS 2017 survey in Oman. The primary survey was approved by the Central Research and Ethics Committee at the Ministry of Health, Oman. (Approval No. 26/2015)^16^. Informed consent was obtained before participating in the study^16^. For this study, permission to obtain secondary data was requested and obtained from the Centre of Research and Study, MOH, Oman, on 27 January 2024.

### Study design

This study was an observational, analytical, cross-sectional study that presented both descriptive and analytical data based on secondary data from the 2017 STEPS survey in Oman.

### Data collection and study instrument

The parent study utilized face-to-face interviews to collect the data, while anthropometric measurements were obtained by trained staff^17^. For the purpose of this study, only 31 variables were included.

### The independent variable

This study included two independent variables: whether participants had been asked about their smoking status and whether they had been advised to quit smoking by a healthcare provider. To assess if participants received advice to quit, they were asked: ‘During any visit to a doctor or other health worker in the past 12 months, were you advised to quit smoking tobacco?’. Responses were grouped as either ‘yes’ or ‘no’. Similarly, for the question ‘During any visit to a doctor or other health worker in the past 12 months, were you asked about your smoking status?’, the answers were divided into ‘yes’ and ‘no’. Participants who reported not visiting any healthcare provider in the past 12 months were excluded from the analysis for both questions.

### The dependent variable

The dependent variable of interest in this study was the quit attempt by tobacco users in the last 12 months in response to the question: ‘During the past 12 months, have you tried to stop smoking?’. Responses were categorized into binary variables of ‘yes’ and ‘no’.

### Sociodemographic and health-related variables

Sociodemographic data (including age, sex, nationality, education level, employment status, marital status, and estimated monthly income) and health-related data (including diabetes mellitus, asthma, high blood pressure or high cholesterol, and body mass index) were examined for their association with quit attempts. Additionally, external environmental factors including seeing warning labels on cigarette packages, seeing health promotion in a newspaper or magazine, watching health promotion on television, hearing health promotion on radio, watching TV shows or films containing cigarette scenes, seeing advertisements in cigarette stores, and supporting a rise in tobacco taxes, were examined for their association with quit attempts.

### Statistical analysis

For descriptive analysis, the categorical variables were presented as frequencies and percentages. Bivariate analyses were performed to examine the associations between sociodemographic variables, health-related variables, wider environmental variables, and quit attempts. Variables with p<0.05 in the bivariate analysis were retained and included in the multivariable analysis. A multivariable binary logistic regression analysis was performed to analyze the association between receiving quit advice from healthcare workers and quit attempts. Adjustments were made for sociodemographic, health-related, and environmental variables. Results were presented as adjusted odds ratios (AORs) and 95% confidence intervals (CIs). The statistical package SPSS version 24 was used to perform the analysis. Two-tailed tests were used, and p<0.05 was considered statistically significant.

## RESULTS

The total number of participants was 6743 (97.9% response rate), with 512 (7.6%) being current tobacco users. Most tobacco users were male (n=504; 98.4%), under <40 years (n=287; 58.6%), married (n=389; 76.0%), employed (n=442; 86.3%), had a secondary or higher level of education (n=350; 68.4%), and had a monthly income of <500 OMR (n=342; 67.5%) (Table 1).

**Table 1:** Sociodemographic characteristics of tobacco users who attempted to quit in the last 12 months based on STEPS survey data, Oman, 2017 (N=512)

*Characteristics*	*Total n (%)*	*Attempt n (%)*	*No attempt n (%)*	p*
**Age** (years)				
<40	287 (58.6)	110 (38.3)	177 (61.7)	0.098
≥40	203 (41.4)	93 (45.8)	110 (54.2)	
**Sex**				
Male	504 (98.4)	209 (41.5)	295 (58.5)	0.074
Female	8 (1.6)	6 (75.0)	2 (25.0)	
**Nationality**				
Omani	232 (45.3)	120 (51.7)	112 (48.3)	**<0.001**
Non-Omani	280 (54.7)	95 (33.9)	185 (66.1)	
**Marital status**				
Unmarried^a^	123 (24.0)	44 (35.8)	79 (64.2)	0.109
Married	389 (76.0)	171 (44.0)	218 (56.0)	
**Education level**				
Illiterate or primary school	162 (31.6)	43 (26.5)	119 (73.5)	**<0.001**
Secondary school or higher	350 (68.4)	172 (49.1)	178 (50.9)	
**Employment status**				
Employed^b^	442 (86.3)	175 (39.6)	267 (60.4)	**0.006**
Unemployed^c^	70 (13.7)	40 (57.1)	30 (42.9)	
**Monthly income** (OMR)				
<500	342 (67.5)	110 (32.2)	232 (67.8)	**<0.001**
≥500	165 (32.5)	101 (61.2)	64 (38.8)	

a Included not married, separated but not divorced, divorced, and widowed.

b Included government employee, non-government employee and self-employed.

c Included students, housewife/housekeeper, retired, unemployed able to work, and unemployed unable to work. OMR: 100 Omani Rials about US$260.

* Pearson chi-squared test; level of significance p<0.05.

In the bivariate analysis, significant associations were observed between quit attempts in the 12 months preceding the survey and several sociodemographic factors, including nationality, education level, employment status, and monthly income (Table 1). Specifically, one-quarter of tobacco users with no formal education or only primary school education reported a quit attempt in the past 12 months (n=43; 26.5%, p<0.001), compared to nearly half of those with secondary or higher levels of education (n=172; 49.1%, p<0.001). Unemployed participants were more likely to attempt quitting than employed participants (n=40; 57.1% vs n=175; 39.6%, p=0.006). Among individuals earning <500 OMR per month, nearly one-third (n=110; 32.2%) attempted to quit (p<0.001), compared to nearly two-thirds (n=101; 61.2%) of those earning ≥500 OMR (p<0.001).

Table 2 shows a descriptive and bivariate analysis of the study population’s attempts to quit based on their tobacco use patterns. One-quarter (n=127; 25.2%) of the study’s participants reported initiating tobacco products before the legal age of 18 years. Nearly two-thirds (n=321; 64.2%) smoked <10 manufactured cigarettes per day. Similarly, the majority reported smoking <10 hand-rolled cigarettes daily (n=437; 86.9%) and using a tobacco pipe <2 times per day (n=447; 89.4%). Most participants (n=484; 95.5%) reported smoking one session of shisha per day. A small proportion of the study population used smokeless tobacco products (n=27; 5.3%) or e-cigarettes (n=2; 2.1%). The univariate analysis revealed no significant association between quit attempts and the frequency or type of tobacco product used.

**Table 2:** Tobacco use patterns of tobacco users who attempted to quit in the last 12 months based on STEPS survey data, Oman, 2017 (N=512)

*Characteristics*	*Total n (%)*	*Attempt n (%)*	*No attempt n (%)*	p*
**Age started to smoke** (years)				
<18	127 (25.2)	60 (47.2)	67 (52.8)	0.210
≥18	377 (74.8)	154 (40.8)	223 (59.2)	
**Manufactured cigarettes per day**				
<10	321 (64.2)	141 (43.9)	180 (56.1)	0.422
≥10	179 (35.8)	72 (40.2)	107 (57.4)	
**Cigarette label**				
Light	137 (39.0)	66 (48.2)	71 (51.8)	**0.019**
Mild	138 (39.3)	51 (37.0)	87 (63.0)	
Low tar	22 (6.3)	9 (40.9)	13 (59.1)	
Unspecified	54 (15.4)	33 (61.1)	21 (38.9)	
**Manufactured hand-rolled cigarettes per day**				
<10	437 (86.9)	179 (41.0)	258 (59.0)	0.248
≥10	66 (13.1)	32 (48.5)	34 (51.5)	
**Pipe full of tobacco per day**				
<2	447 (89.4)	183 (40.9)	264 (59.1)	0.384
≥2	53 (10.6)	25 (47.2)	28 (52.8)	
**Cigars/cheroots/cigarillos per day**				
<10	411 (82.0)	175 (42.6)	236 (57.4)	0.951
≥10	90 (18.0)	38 (42.2)	52 (57.8)	
**Shisha sessions per day**				
<1	484 (95.5)	203 (41.9)	281 (58.1)	0.884
≥1	23 (4.5)	10 (43.5)	13 (56.5)	
**Currently use any smokeless tobacco product**				
Yes	27 (5.3)	10 (37.0)	17 (63.0)	0.592
No	485 (94.7)	205 (42.3)	280 (57.7)	
**Currently use e-cigarettes**				
Yes	2 (1.3)	1 (50.0)	1 (50.0)	0.943
No	158 (98.7)	83 (52.5)	75 (47.5)	

* Pearson chi-squared test; level of significance p<0.05.

Table 3 displays the descriptive and bivariate analysis of quit attempts based on health-related characteristics. The health profiles of tobacco users revealed that most of them did not drink alcohol (n=482; 94.1%), and were non-asthmatic (n=496; 96.1%), normotensive (n=337; 89.6%), and non-diabetic (n=474; 92.6%). Over one-third of the study participants (n=207; 41.4%) had a normal BMI <25 (kg/m^2^). None of these health-related characteristics had a significant association with an attempt to quit.

**Table 3:** Health-related characteristics of tobacco users who attempted to quit in the last 12 month based on STEPS survey data, Oman, 2017 (N=512)

*Characteristics*	*Total n (%)*	*Attempt n (%)*	*No attempt n (%)*	p*
**Alcohol consumption**				
Yes	30 (5.9)	16 (53.3)	14 (46.7)	0.195
No	482 (94.1)	199 (41.3)	283 (58.7)	
**Asthma**				
Yes	16 (3.1)	10 (62.5)	6 (37.5)	0.091
No	496 (96.8)	205 (41.3)	291 (58.7)	
**Hypertension**				
Yes	39 (10.4)	23 (59.0)	16 (41.0)	0.063
No	337 (89.6)	146 (43.3)	191 (56.7)	
**Dyslipidemia**				
Yes	25 (15.2)	13 (52.0)	12 (48.0)	0.494
No	139 (84.8)	62 (44.6)	77 (55.4)	
**Diabetes mellitus**				
Yes	38 (7.4)	21 (55.3)	17 (44.7)	0.085
No	474 (92.6)	194 (40.9)	280 (59.1)	
**BMI**				
<25	207 (41.4)	77 (37.2)	130 (62.8)	0.067
≥25	293 (58.6)	133 (45.4)	160 (54.6)	
**HCW asked about smoking status**				
Yes	207 (48.1)	92 (44.4)	115 (55.6)	0.195
No	223 (51.9)	84 (37.7)	139 (62.3)	
**Advised to quit smoking by HCW**				
Yes	167 (41.4)	82 (49.1)	85 (50.9)	**0.020**
No	236 (58.6)	83 (35.2)	153 (64.8)	

BMI: body mass index (kg/m^2^). HCW: healthcare workers.

* Pearson chi-squared test; level of significance p<0.05.

Almost half of the study participants (n=207; 48.1%) reported being asked about their smoking status by a healthcare provider; however, this was not associated with an increase in attempts to quit. On the other hand, over one-third (n=167; 41.4%) of the study population reported receiving advice to quit smoking. Among those who received advice to quit, nearly half (n=82; 49.1%) had attempted to quit in the last 12 months, which was statistically significant (p=0.02).

Table 4 describes the external factors that may influence quit attempts in the study population. Watching tobacco-related health warning messages on television was significantly associated with quit attempts, with almost half of those exposed (n=125; 46.8%) reporting an attempt to quit. However, exposure to similar messages in newspapers, magazines, or on the radio showed no significant association with quit attempts.

**Table 4:** External factors that influence quitting attempts among tobacco users during the past 30 days, based on STEPS survey data, Oman, 2017 (N=512)

*Characteristics*	*Total n (%)*	*Attempt n (%)*	*No attempt n (%)*	p*
**Seeing warning labels on cigarette packages**				
Yes	352 (71.8)	155 (44.0)	197 (56.0)	0.115
No	138 (28.2)	50 (36.2)	88 (63.8)	
**Noticed health warning in a newspaper or magazine**				
Yes	225 (46.2)	104 (46.2)	121 (53.8)	0.172
No	262 (53.8)	105 (40.1)	157 (59.9)	
**Noticed health warning on television**				
Yes	267 (54.6)	125 (46.8)	142 (53.2)	**0.046**
No	222 (45.4)	84 (37.8)	138 (62.2)	
**Noticed health warning on radio**				
Yes	158 (32.6)	68 (43.0)	90 (57.0)	0.912
No	327 (67.4)	139 (42.5)	188 (57.5)	
**Watch actors with smoking scenes**				
Yes	303 (66.3)	143 (47.2)	160 (52.8)	**0.019**
No	154 (33.7)	49 (31.2)	105 (68.2)	
**Seeing advertisements in cigarette store**				
Yes	64 (13.0)	31 (48.4)	33 (51.6)	0.409
No	427 (87.0)	177 (41.5)	250 (58.5)	
**Favoring raise in tobacco taxes**				
Yes	267 (58.3)	133 (49.8)	134 (50.2)	**<0.001**
No	191 (41.7)	72 (37.7)	119 (62.3)	
**Having one smoke at home**				
Yes	235 (45.9)	92 (39.1)	143 (60.9)	0.230
No	277 (54.1)	123 (44.4)	154 (55.6)	
**Having one smoke in closed area in your workplace**				
Yes	86 (22.0)	36 (41.9)	50 (58.1)	0.971
No	305 (78.0)	127 (41.6)	178 (58.4)	

Hosmer-Lemeshow test, HL=0.552; Nagelkerke R^2^=0.247.

* Level of significance p<0.05.

Two-thirds (n=303; 66.3%) of study participants had seen TV shows or films with smoking scenes; with nearly half (n=143; 47.2%) having attempted to quit (p=0.019). No significant association was found between quit attempts and exposure to tobacco advertisements in stores selling tobacco products. Among smokers who reported having someone smoke at home, only one-third (n=192; 39.1%) attempted to quit; however, this association was not statistically significant. Similarly, no significant association was observed between a quit attempt and smoking in an enclosed workplace.

Table 5 shows a binary logistic regression analysis of the association between sociodemographic, health-related, and external variables and quit attempts. After controlling for other variables, participants who received quit advice from healthcare providers were 3.13 times more likely to attempt quitting than those who did not (AOR=3.13; 95% CI: 1.46–6.71, p=0.003). Participants with secondary or higher level of education were 3.35 times more likely to attempt quitting compared to those with low level of education (AOR=3.35; 95% CI 1.67–6.72, p<0.001). Furthermore, tobacco users aged ≥40 years were 77.0% more likely to attempt quitting than those aged <40 years (AOR=1.77; 95% CI: 1.00–3.13, p=0.048). Tobacco users with monthly incomes ≥500 OMR were 2.21 times more likely to attempt quitting than those with lower monthly incomes (AOR=2.21; 95% CI: 1.24–3.93, p<0.007). Unemployed tobacco users were 3.38 times more likely to quit compared to employed tobacco users (AOR=3.38; 95% CI: 1.53–7.47, p<0.003). Those who watched a tobacco-related health warning on television were 2.06 times more likely to attempt to quit compared to those who had not (AOR=2.06, 95% CI: 1.02–4.12, p<0.043).

**Table 5:** Logistic regression analysis exploring the association between quitting attempts and different sociodemographic and health-related and external variables, based on STEPS survey data, Oman, 2017 (N=512)

*Variables*	*AOR*	*95% CI*	p*
**Ask about tobacco smoking**			
No ®	1		
Yes	0.6	0.29–1.24	0.168
**Advised to quit smoking by HCW**			
No ®	1		
Yes	3.13	1.46–6.71	**0.003**
**Age** (years)			
<40 ®	1		
≥40	1.77	1.00–3.13	**0.048**
**Nationality**			
Omani ®	1		
Non-Omani	0.92	0.51–1.65	0.768
**Marital status**			
Unmarried^a^ ®	1		
Married	0.913	0.46–1.81	0.795
**Education level**			
Illiterate or primary school ®	1		
Secondary school or higher	3.35	1.67–6.72	**<0.001**
**Employment status**			
Employed^b^ ®	1		
Unemployed^c^	3.38	1.53–7.47	**<0.003**
**Monthly income** (OMR)			
<500 ®	1		
≥500	2.21	1.24–3.93	**0.007**
**Noticed health warning on cigarette packaged**			
No ®	1		
Yes	1.26	0.68–2.33	0.456
**Notice health warning in newspaper or magazine^d^**			
No ®	1		
Yes	0.537	0.258–1.12	0.096
**Noticed health warning on television^d^**			
No ®	1		
Yes	2.06	1.02–4.12	**0.043**
**Noticed health warning on radio^d^**			
No ®	1		
Yes	0.81	0.39–1.68	0.575
**Favoring increased tobacco taxes**			
No ®	1		
Yes	1.67	0.96–2.885	0.069
**Watched actors with smoking scenes^d^**			
No ®	1		
Yes	1.62	0.86–3.05	0.135
**Advertisement of tobacco in stored**			
No ®	1		
Yes	0.73	0.35–1.51	0.393
**Age started smoking** (years)			
<18 ®	1		
≥18	1.07	0.58–1.96	0.830

AOR: adjusted odds ratio.

a Included not married, separated but not divorced, divorced, and widowed.

b Included government employee, non-government employee and self-employed;

c Included students, housewife/housemaker, retired, unemployed able to work, and unemployed unable to work.

d During the past 30 days from the survey. Hosmer-Lemeshow test, HL=0.552; Nagelkerke R2=0.247. ® Reference categories.

* Level of significance p<0.05.

## DISCUSSION

This study highlights various factors that may influence a quit attempt, using a nationally representative sample from the 2017 STEPS survey. Quit attempts were more common among Omani participants, those with a secondary school or higher level of education, those who were unemployed, and those who earned ≥500 OMR per month. Receiving advice to quit tobacco products from healthcare providers was significantly associated with an increase in quit attempts. No significant association was found between the type of tobacco used or participants’ medical background and the quit attempt in the study cohort.

### Sociodemographic disparities and quit attempts

The current study showed that quit attempts were significantly and independently high among older age groups, employed individuals, those with higher levels of education, and those with a monthly income of ≥500 OMR, both in bivariate and multivariate analyses, and this was consistent with existing literature^17-20^. In contrast to the existing literature^19^, unemployed tobacco users were more likely to attempt quitting than employed tobacco users. The current study showed that the older age group was more likely to attempt quitting. While some studies showed comparable findings^7^, others demonstrated no significant association^21,22^. The current study showed that female tobacco users were more likely to attempt to quit compared to male tobacco users; however, this was not statistically significant. The study did not assess the effect of area of residence due to the absence of this variable in the parent survey. However, studies have found a significant association between residential area and quit attempts^18^.

Several reasons could explain the sociodemographic disparity in attempts to quit. First, access to cessation services and perceptions of their effectiveness may influence intention and subsequently attempts to quit^17^. Second, a person’s intrinsic motivation to quit is a powerful predictor of an attempt to quit, as is the rule of family or peer pressure^8^. Third, differences in quit attempt rates across studies may be attributed to variations in study design, country-specific characteristics, national tobacco control policies, and the timing of data collection^17^. Understanding these factors can help identify where disparities exist and how they can be effectively addressed.

### Quit advice provided by healthcare providers and quit attempts

When adjusting for sociodemographic, health-related, and external factors, receiving advice by healthcare workers to quit tobacco products was associated with a 3.13 times increase chance to attempt quitting (AOR=3.13; 95% CI: 1.46–6.71, p=0.003), and this is consistent with existing literature^22–25^. However, the STEPS 2017 survey did not specifically explore the duration of the quit advice or the professional background of the provider, both of which could influence its effectiveness. Approximately 40% of tobacco users make a quit attempt in response to advice from a general practitioner, though such interventions are often limited by time constraints^26^. The impact of healthcare providers in providing quit advice is not limited to physicians; professionals from various disciplines can also play an effective role, provided they receive appropriate training^26^.

Behavioral interventions, such as brief advice, individual and group support, telephone counseling, self-help material, and text messages, have varying effectiveness in helping people quit tobacco. Group therapy and individual support are better than self-help and less intensive interventions, with the chance of quitting increasing by 50% to 130%^27^. However, there is insufficient evidence to determine whether group therapy is more effective or cost-effective than intensive individual counseling^17^. On the other hand, individual-based support can increase the chance of quitting by 40% to 80% compared to minimal support^28^. Telephone counseling increases the chance of quitting smoking, regardless of whether the patient is motivated to quit and/or receiving other tobacco cessation treatment. However, proactive telephone counseling is considered superior to reactive telephone counseling in terms of boosting the quit rate^29^. Additionally, smokers who received 3–5 calls were more likely to quit than those who received only one call^29^. Minimal evidence showed that motivational interviewing may or may not help people to quit compared with no intervention or compared with other type of behavioral intervention^30^. Automated text messagebased tobacco cessation interventions result in greater quit rates than minimal support^31^.

Several challenges can interfere with providing advice to quit tobacco products by healthcare providers. The primary constraint is the limited time and lack of personalized support necessary to effectively deliver evidence-based tobacco cessation advice^32^. Furthermore, healthcare providers frequently possess insufficient knowledge about the harms of tobacco products and lack adequate training to provide effective tobacco cessation services. Tobacco use status is not routinely recorded during each clinical encounters. Tobacco cessation advice is often limited to doctors and nurses within primary healthcare settings^33^, with no clear plan to extend it to other disciplines from secondary or tertiary healthcare. Addressing these gaps could significantly enhance healthcare providers’ capacity to deliver effective cessation interventions at all levels of healthcare.

### Health-related factors and quit attempts

The current study found no significant association between having a long-standing medical illness and attempting to quit. The low prevalence of tobacco use in this study group may explain the absence of association between medical background and attempts to quit. However, existing literature has shown that poor health is one of the motivators for tobacco users to quit^34^. A study in Poland identified general health concerns (57%), personal health issues (32%), and social reasons (32%) as key motivators to quit^34^.

The current study did not show significant association between exposure to secondhand smoke (at home, in the workplace, or while visiting a healthcare facility) and quit attempts, which was inconsistent to the existing literature^26^. The current study found no significant association between age of initiation and attempts to quit. Other studies showed that older age at initiation was associated with increased intention to quit^21^.

### External factors and attempt to quit

The current study explored external factors that may influence quit attempts. Nearly half (44.0%) of those who saw warning labels on cigarette packages attempted to quit, however, this was not statistically significant (p=0.115). Existing literature found that those who saw a health warning on plain packaging were more likely to attempt to quit^25^. Furthermore, a study in Australian adults found that plain packaging with larger graphic health warnings increased quitting intention and quit attempts among adult cigarette smokers^35^. The current study found no significant association between quit attempts and health warning in plain packaging, possibly due to the absence of plain packaging policy on all manufactured tobacco products at the time the primary survey was conducted.

On the other hand, noticing a tobacco-related health warning on TV was associated with quit attempt; with 46.8% who saw this health promotion attempted to quit, p=0.046. No significant associations were observed with warnings seen in newspapers or magazines or heard on the radio.

### Implications

Article 14 of the WHO Framework Convention on Tobacco Control (FCTC) recommends that any tobacco cessation program include at least three types of cessation supports: tobacco cessation medication, cessation counseling in a primary care setting, and toll-free Quitline^13^. These should be part of comprehensive, multisectoral tobacco control initiative to ensure effectiveness. Creating an enabling environment that empowers healthcare providers to deliver quit advice at every clinical encounter, by addressing barriers at the national, healthcare system, and individual levels, is essential. Several recommendations can be made from this study.

First, integrate cessation services at all levels of the healthcare system. The electronic record system already has a specialized tobacco cessation module; however, national coordination is required to ensure effective implementation at all healthcare levels. This will guarantee that all tobacco users receive a comprehensive, evidence-based treatment plan to support them throughout their quit journey.

Second, while ongoing tobacco cessation training is being provided at the primary care level to build the capacity of healthcare providers in various healthcare sectors, it is only limited to doctors and nurses. There is no clear plan to expand the service to other sectors. Thus, establishing national training coordination is crucial to broaden training efforts and equip frontline healthcare workers across various disciplines to deliver tobacco cessation support as part of their routine responsibilities.

Third, implement brief advice at every clinical encounter. A well-defined referral pathway should be established for individuals who express a willingness to quit tobacco products^1^. Introducing a Quitline service would significantly aid quit attempts.

Fourth, the availability of tobacco medication is a major deterrent to enabling tobacco users to quit, and even when it is available, the 100% taxes on nicotine replacement therapy, make the attempt even more difficult. Thus, it is crucial to make these medications available in the first instance and affordable to those who wish to quit.

Fifth, tobacco cessation program should be supported by population-based interventions, such as multi-sectoral tobacco control policies, as part of FCTC’s demand reduction measures to reduce tobacco uptake while increasing quit attempts and rates. Among these measures are: 1) monitoring tobacco use and prevention policies; 2) protecting people from exposure to secondhand smoke; 3) offering help to quit tobacco use; 4) warning about the dangers of tobacco use; 5) enforcing bans on tobacco advertising, promotion, and sponsorship; and 6) raising taxes on tobacco products. While Oman has made progress, by introducing 100% retail taxation on cigarette products and plain packaging—more can be done, such as ‘complete’ banning of indoor smoking areas^12^. Implementing a comprehensive cessation policy can reduce smoking prevalence at 5 years by 3.5–9% and nearly avert 9000 deaths at 40 years. However, having a comprehensive tobacco control policy combined, can reduce smoking prevalence by 38% and 48% at 5 and 40 years and avert 65000 deaths at 40 years^12^.

Lastly, implementing a nationwide tobacco control surveillance and monitoring system that routinely tracks tobacco use and assesses the effectiveness of policies is essential for identifying and addressing gaps in tobacco control efforts.

### Strengths and limitations

This is the first study to examine the association between receiving advice by a healthcare provider and quit attempts in a nationally representative sample in Oman. The overall response rate for the 2017 STEPS survey was significantly high (97.9%), making the data more generalizable to the entire population. Data collection was done by professional personnel; therefore, the risks of misinterpretation or missing data were minimized to some extent. Finally, the study adjusted for a number of sociodemographic, health-related, and external variables.

Nonetheless, there are some limitations to this study. The major limitation is the cross-sectional design, which precludes the inference of a causal relationship between advice from a healthcare provider and a quit attempt. Furthermore, this study was susceptible to recall bias, response bias, and social desirability bias. The study did not examine the geographical distribution of tobacco users, previous quit attempts, withdrawal symptoms, or quit rates, as these data were not available in the primary survey. The current study did not stratify participants by sex to identify variations in the association between advice to quit and quit attempts, as the prevalence of smoking among women was extremely low.

## CONCLUSIONS

This is the first analytical, cross-sectional study to use STEPS 2017 data to examine the factors that influence quit attempts among tobacco users in Oman. The study identified a socioeconomic disparity in quit attempts, with higher quit attempts observed among older tobacco users, those with higher level of education, employed individuals, and those with higher monthly income. These disparities should be addressed when developing tobacco cessation strategies as part of a broader tobacco control initiative. Furthermore, the current study found that providing quit advice by a healthcare providers was associated with an increased likelihood of attempting to quit. Furthermore, the current study showed that providing advice by healthcare providers to quit tobacco products was associated with an increased likelihood of attempting to quit. Establishing national coordination, along with continuous monitoring, is critical for the successful implementation of the tobacco cessation program. Improved access to cessation support for disadvantaged tobacco users is also necessary. Further research is needed to investigate the broader determinants of quit attempts and quit rates.

## Data Availability

The data used in this study are available upon request from the Centre of Research and Study, Ministry of Health, Oman.
